# Supplementary material for: Optimizing Primary Human Salivary Stem/Progenitor Cells for Tissue Engineering Applications
Source: bioRxiv. 2026 May 13:2026.05.12.724408. Preprint. [Version 1] doi: 10.64898/2026.05.12.724408 (PMC13192770; doi:10.64898/2026.05.12.724408)
Supplement: Supplement 1 [file media-1.pdf]

Supplementary Table 1. *Antibodies used for Immunocytochemistry/Flow Cytometry*

Antibodies	*Manufacturer*	*Dilution*	*Cat. no.*
CD44-FITC	Miltenyi Inc	1:400	130-113-896
CD133-PE	Miltenyi Inc	1:400	130-113-748
Anti-Cytokeratin 14 (K14)	Abcam	1:200	Ab7800
Purified Anti-Cytokeratin 5 (K5)	BioLegend	1:500	905504
DAPI (4′,6-diamidino-2-phenylindole)	Invitrogen	1:500	D3571
Fibroblast-APC	Miltenyi Inc	1:100	130-100-133
Anti-fibroblasts Antibody (TE-7)	Novus Biologicals	1:100	NBP2-50082
LIVE/DEAD™ Fixable Blue Dead Cell Stain Kit	Invitrogen	1:40	L34962A

Abbreviations: FITC, fluorescein isothiocyanate; PE, phycoerythrin; APC, allophycocyanin.
